# Refining mutanome-based individualised immunotherapy of melanoma using artificial intelligence

**DOI:** 10.1186/s40001-023-01625-2

**Published:** 2024-01-05

**Authors:** Farida Zakariya, Fatma K. Salem, Abdulwhhab Abu Alamrain, Vivek Sanker, Zainab G. Abdelazeem, Mohamed Hosameldin, Joecelyn Kirani Tan, Rachel Howard, Helen Huang, Wireko Andrew Awuah

**Affiliations:** 1 Faculty of Pharmaceutical Sciences, Ahmadu Bello University, Zaria, Nigeria; 2 Division of Experimental Medicine, Faculty of Medicine and Health Sciences, McGill University, Montreal, Canada; 3 Faculty of Veterinary Medicine, South Valley University, Qena, 83523 Egypt; 4 Faculty of Medicine, Al-Quds University, Abu Dis, Palestine; 5 Research Assistant, Dept. Of Neurosurgery, Trivandrum Medical College, Trivandrum, India; 6 Division of Molecular Biology, Department of Zoology, Faculty of Science, Alexandria University, Alexandria, Egypt; 7 Faculty of Pharmacy, Zagazig University, Sharqia, Egypt; 8 Faculty of Medicine, University of St Andrews, St Andrews, Scotland, UK; 9 School of Clinical Medicine, University of Cambridge, Cambridge, England; 10 Faculty of Medicine and Health Science, Royal College of Surgeons in Ireland, Dublin, Ireland; 11 Medical Institute, Sumy State University, Zamonstanksya 7, Sumy, 40007 Ukraine

**Keywords:** Mutanome, Melanoma, Immunotherapy, Artificial intelligence

## Abstract

Using the particular nature of melanoma mutanomes to develop medicines that activate the immune system against specific mutations is a game changer in immunotherapy individualisation. It offers a viable solution to the recent rise in resistance to accessible immunotherapy alternatives, with some patients demonstrating innate resistance to these drugs despite past sensitisation to these agents. However, various obstacles stand in the way of this method, most notably the practicality of sequencing each patient's mutanome, selecting immunotherapy targets, and manufacturing specific medications on a large scale. With the robustness and advancement in research techniques, artificial intelligence (AI) is a potential tool that can help refine the mutanome-based immunotherapy for melanoma. Mutanome-based techniques are being employed in the development of immune-stimulating vaccines, improving current options such as adoptive cell treatment, and simplifying immunotherapy responses. Although the use of AI in these approaches is limited by data paucity, cost implications, flaws in AI inference capabilities, and the incapacity of AI to apply data to a broad population, its potential for improving immunotherapy is limitless. Thus, in-depth research on how AI might help the individualisation of immunotherapy utilising knowledge of mutanomes is critical, and this should be at the forefront of melanoma management.

## Introduction

Melanoma is a rare type of skin tumour, accounting for 1.1% of cancer deaths per year [1]. The annual incidence of melanoma has rapidly increased worldwide [2]. However, there has been a reduction in the mortality rate due to advancements in immunotherapy [3]. There are significant regional melanoma variations around the world that are attributed to racial skin phenotypes and sun exposure [4], which is the most implicated cause of melanoma globally [5]. Moreover, melanoma occurs mainly in young and middle-aged people, with an increasing incidence after age 25 and decreasing after 50, particularly in females [6].

The pathophysiology of melanoma involves mutations in genes regulating proteins, tight junctions, the cell cycle, deoxyribonucleic acid (DNA) damage, and remodelling of chromatin related to the melanocytes [5]. *BRAF* and *NRAS* are the most implicated genes, contributing 54.4% and 30.7% due to mutations at the V600 codon and Q61 codon, respectively [5]. Various treatment approaches have been developed in the management of melanoma, and these approaches have been continually refined, with new modalities added to better streamline the available options and increase survival rates. Available treatment options include surgical excision, chemotherapy, targeted therapy using *BRAF*, *NRAS*, and *C-Kit* inhibitors, radiation, and immunotherapy [1].

With the advent of immunotherapy, the median survival rate of advanced melanoma has improved from 9 months to 6 years [7]. Due to the variability in mutations in melanoma, knowledge of the status of individual mutations can help in patient stratification and aid targeted immunotherapy. In recent times, understanding individual mutations known as mutanomes has gained traction as a potential means for managing advanced cancers refractory to known therapies [8]. Advancement in artificial intelligence (AI) has sparked the debate that rapid sequencing of the mutanome and streamlining therapy options that trigger the immune system to target individual mutations will significantly improve therapy outcomes [9]. Particularly in melanoma, where adoptive cell therapy is proving to be a promising option for mutation-targeted immunotherapy, interest in artificial intelligence for refining this approach is increasing. One of the main reasons why AI is becoming more prominent in refining available immunotherapy options for melanoma is the increasing rate of reported resistance and refraction experienced by patients [10]. It is becoming apparent that finding approaches that utilise the differences in individual mutations and targeting these mutations on a personalised basis will help reduce the rate at which treatment failure occurs. However, due to the heterogeneity of melanoma, developing vaccines or personalising therapy for each patient is a tedious and expensive endeavour. Thus, this review seeks to highlight the applicability of AI in refining melanoma immunotherapy through exploring the differences in individual mutations**.**

## Methodology

This narrative review systematically investigates the potential applications of artificial intelligence in advancing research on mutanome-based individualisation of immunotherapy for melanoma management. Employing a rigorous methodology, the review encompasses a diverse range of study designs, including observational, case–control, cohort, and randomised controlled trials, with consideration for both paediatric and adult populations. The inclusion criteria, meticulously formulated, strictly adhere to English-language publications, spanning the period from 2001 to 2023 to align with contemporary practices.

To ensure a thorough exploration of the subject matter, the literature search utilised reputable databases such as ScienceDirect and PubMed. A thoughtfully selected set of search terms, including “mutanome”, “melanoma”, “immunotherapy”, and “artificial intelligence”, tailored the search to the specific focus of interest. Additionally, a manual search enriched the review by identifying references related to recently published, disease-specific reviews. Notably, stand-alone abstracts and unpublished studies were deliberately excluded.

Through this comprehensive and meticulous approach, the review aims to provide a scholarly assessment of the integration of AI technology in refining current research on mutanome-based individualisation of immunotherapy for melanoma management. The employed methodology is summarised in Table 1 for clarity and reference.

**Table 1:** Summary of the methodology employed in the study

Methodology steps	Description
Literature search	ScienceDirect, PubMed
Inclusion criteria	Full-text articles published in English
	Various study designs, such as observational, case–control, cohort, cross-sectional, and randomised controlled trials
	Studies involving paediatric and adult populations
	Studies published between 2001 and 2023
Exclusion criteria	Stand-alone abstracts and unpublished studies. Non English Studies
Search terms	“Mutanome”, “melanoma”, “immunotherapy” and “artificial intelligence”
Additional search	A manual search was conducted to find references for recently published, procedure-specific reviews

## Melanoma

### Aetiology of malignant melanoma

Melanoma is caused by multifactorial interactions between the body and the environment [11]. Melanoma is mainly derived from the accumulation of several mutations in melanocyte genes. *NRAS*, *BRAF*, and *PTEN* are some of the most significant genes in the development of melanoma [12–15, 17, 17]. There are also various genes for which mutations can be inherited, resulting in hereditary melanoma, such as *CDKN2A*, *CDK4*, *TP53*, *BRCA1*, *BRCA2*, and *PTEN* [18]*.*

Environmental factors such as exposure to ultraviolet rays, which is considered the leading risk factor for melanoma [19] can disrupt melanocytes either directly by causing oxidative stress [20, 21] or indirectly by causing several mutations that induce carcinogenesis [22, 23]. Moreover, the risk of developing melanoma rises substantially with overexposure to sun and ultraviolet (UV) rays in addition to recurring sunburns, particularly in younger age groups [24, 25]. It is also influenced by the skin phototype, as among the six skin phototypes, those with fair skin, blue eyes, and blond or red hair (Phototypes I and II) are the most vulnerable to developing skin melanoma due to their high sensitivity to UVB rays [26]*.*

Another environmental factor is the geographical location, as melanoma incidence shows various rates in different regions, with the highest incidence rates in Australia and New Zealand [27]*.* More interestingly, it was found that acral melanoma on the hands' palms and the feet soles is more prevalent in people working with herbicides such as dichlorprop, atrazine, propanil, and paraquat, and it has a higher incidence in those using these herbicides at home than in those who do not [28]*.* Also, the susceptibility to skin melanoma is significantly influenced by the status of immunity, as immunosuppressive diseases such as Acquired Immunodeficiency Syndrome (AIDS) increase the risk of developing skin melanoma due to the inability of compromised immunity to effectively protect the body against the formation and development of solid tumours [29, 30]*.*

### Clinical manifestation of melanoma

Melanoma can manifest in different forms depending on the primary location of melanocyte transformation. They broadly occur from mutations in the skin melanocytes known as cutaneous melanoma; the iris, choroid, and ciliary body melanocytes collectively referred to as uveal melanomas; and the mucosal melanocytes leading to mucosal melanoma [31]. Of the three, cutaneous melanoma is the most predominant, accounting for 91.2% of all melanoma cases. The National Comprehensive Cancer Network (NCCN) set a new standard in 2017 to classify cutaneous melanoma into 4 types: chronic sun damage (CSD), non-chronic sun damage (non-CSD), acral, and mucosal melanomas. CSD-melanomas are asymmetric, flat, yellowish-brown, brown, or black macules with irregular borders. Non-CSD melanomas are divided mainly into superficial spreading melanoma (SSM), which begins as an asymptomatic tan to black macules that then grow radically, and nodular melanoma (NM), which commonly appears as blue or black, but sometimes pink to red nodules that lack Asymmetry, Border, Colour, Diameter and Evolving (ABCDE) features and can turn into elevated nodules, ulcers, or bleeding. Acral melanoma (AM) is characterised by irregular pigmentation, parallel ridges, and multicomponent lesions on hairless areas such as the palms, fingernails, soles, and toenails. Mucosal melanoma can be found in the lips, eyelids, oral cavity, intestinal mucosa, vulva, and many other sites. It appears as structureless, grey areas in early dermoscopic diagnosis and as lesions with a multicomponent pattern in advanced dermoscopic diagnosis [32].

### Limitations and challenges in the management of malignant melanoma

The surgical removal option is primarily used for localised melanoma [33]. It can be used in some metastatic melanoma cases as well, but it is not considered to be curative, and other treatment options are still needed, such as chemotherapy. Although chemotherapy was the only curative option for metastatic melanoma until recently, its usage has decreased since the appearance of immunotherapies and targeted therapies [34]. To treat melanoma, numerous targeted therapies have been developed, among which the *BRAF* inhibitors vemurafenib and dabrafenib are the most promising [35, 36]. Despite their high efficacy, secondary resistance within a short time has been observed in most of the patients with *BRAF*-mutated melanomas [35–37]. Because of the high expense and severe side effects of the current treatments, research is still ongoing to overcome the limitations and complications, improve safety, and find other drug options [34].

### The use of targeted therapy in malignant melanoma

A variety of cancer inhibitors are used in targeted therapy, including mitogen-activated protein kinase (MEK) inhibitors (trametinib), *BRAF* inhibitors (vemurafenib and dabrafenib), cyclin-dependent kinase (CDK) inhibitors (ribociclib, abemaciclib, and palbociclib), and c-Kit inhibitors (imatinib) [38]*.* Trametinib is a monotherapy-approved MEK1/MEK2 inhibitor used to treat *BRAF* V600-mutant metastatic melanoma [39]*.* Although vemurafenib is a *BRAF* mutant inhibitor with high selectivity and efficacy against metastatic melanoma with *BRAF* V600 and non-V600E mutations [38], treatment resistance develops in most patients [40]*.* Dabrafenib is a subsequent-generation *BRAF* mutant inhibitor. The Food and Drug Administration (FDA) approved it for the treatment of unresectable or metastatic *BRAF* V600E-mutated melanomas [41, 42]. Ribociclib, abemaciclib, and palbociclib are a new class of specific CDK4/6 inhibitors that are more effective and have fewer side effects [38]*.* Imatinib is a c-Kit inhibitor found to be effective against c-Kit-mutated metastatic melanomas [43]*.*

### Immunotherapy options available for malignant melanoma

Substantial advances have been made in immunotherapy treatments for metastatic melanoma over the last three decades. Cancer vaccines, adoptive cell therapies, and immunomodulatory approaches are the primary three types of immunotherapy options [34]. Interleukin-2 treatment was one of the first immune therapies for metastatic melanoma [35, 44]. Unfortunately, it was found to be highly toxic [34]. Cancer vaccines are therapeutic vaccines designed to stimulate the immune system against cancer cells. Due to the various evasion mechanisms cancer cells have, creating these vaccines has been challenging, so the early vaccines were not effective, and none have been approved for clinical application yet [45–47].

**Fig. 1:**
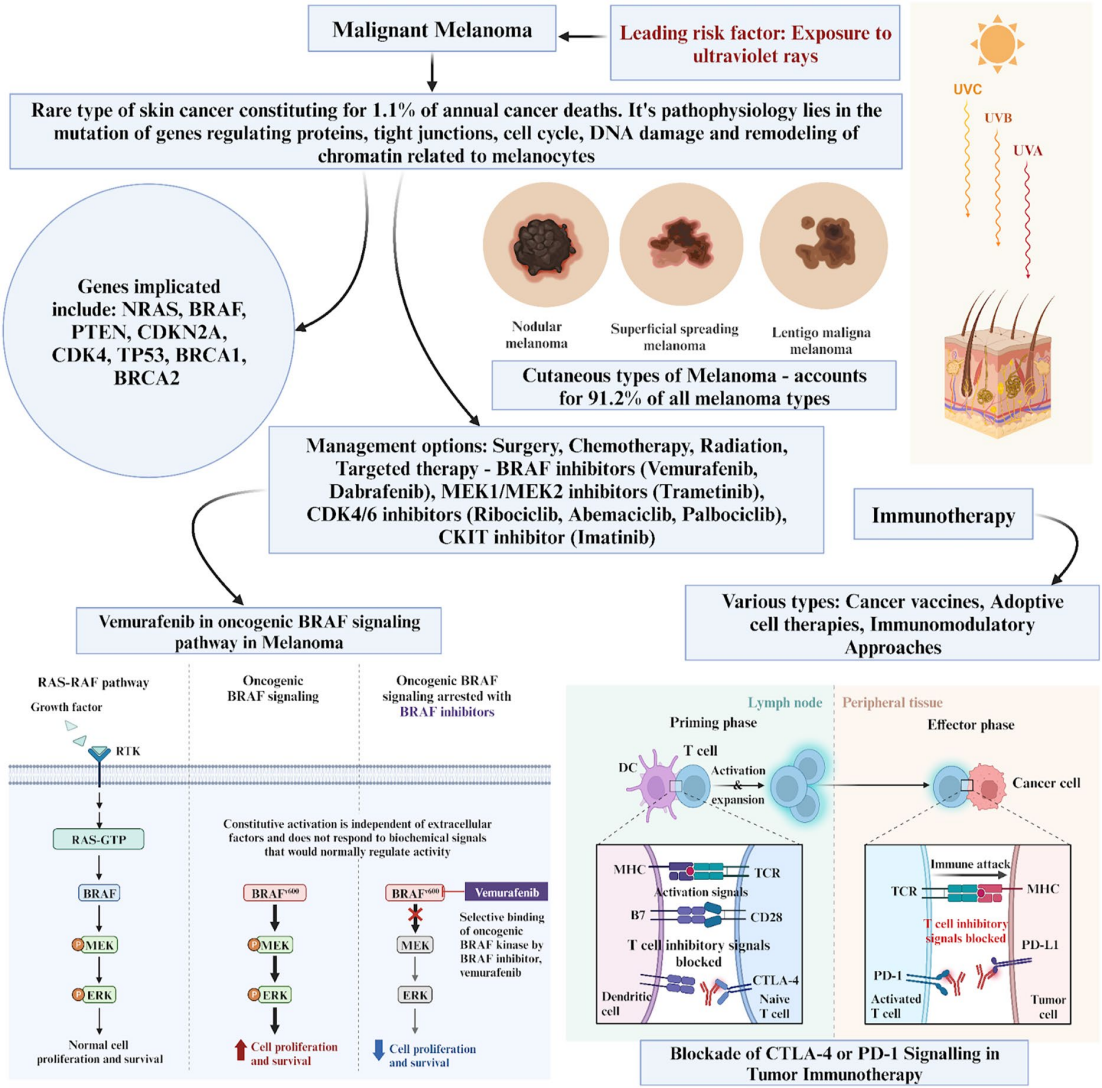
Overview of malignant melanoma along with its new therapeutic targets. CTLA-4, cytotoxic T-lymphocyte associated protein-4; PD-1, programmed cell death protein 1; TCR, T cell receptor; PDL-1, programmed death ligand 1; MHC, major histocompatibility complex; DC, dendritic cell; BRAF, v-Raf murine sarcoma viral oncogene homolog B1; MEK, mitogen-activated protein kinase; ERK, extracellular signal-regulated kinase; RAF, rapidly activated fibrosarcoma; RAS, rat sarcoma; RTK, receptor tyrosine kinase; GTP, guanosine triphosphate; NRAS, neuroblastoma RAS viral oncogene homolog; PTEN, phosphatase and tensin homolog; TP53, tumour protein 53; BRCA, breast cancer gene; CDK, Cyclin dependent kinase

Up to date, the most effective treatment is immune checkpoint inhibitors [44, 48, 49]. Antibodies against the immune checkpoint receptors, such as programmed cell death protein 1 (PD-1), PD-1 ligand (PD-L1/2), and cytotoxic T-lymphocyte-associated protein 4 (CTLA-4), can be used to counteract the immune checkpoint modulation in melanoma. These antibodies disrupt binding to the corresponding ligands and tolerance signals, ultimately leading to the activation of the immune system [49–52]. The anti-CTLA-4 antibody ipilimumab and the anti-PD-1 antibodies nivolumab and pembrolizumab are currently the approved immune checkpoint inhibitor drugs for melanoma treatment [51]. Despite the benefits of checkpoint inhibitors, they have serious side effects mainly related to immunity because they inhibit the tolerance of immune mechanisms [53, 54]. Corticosteroids can neutralise their toxicity in some cases, but others continue to struggle with these side effects. In addition, a majority of patients still show no response, and others may even acquire secondary resistance [34, 55]. Overview of malignant melanoma with its newer therapeutic targets is summarised in Fig. 1.

## Mutanome-based individualised immunotherapy for malignant melanoma

**Fig. 2:**
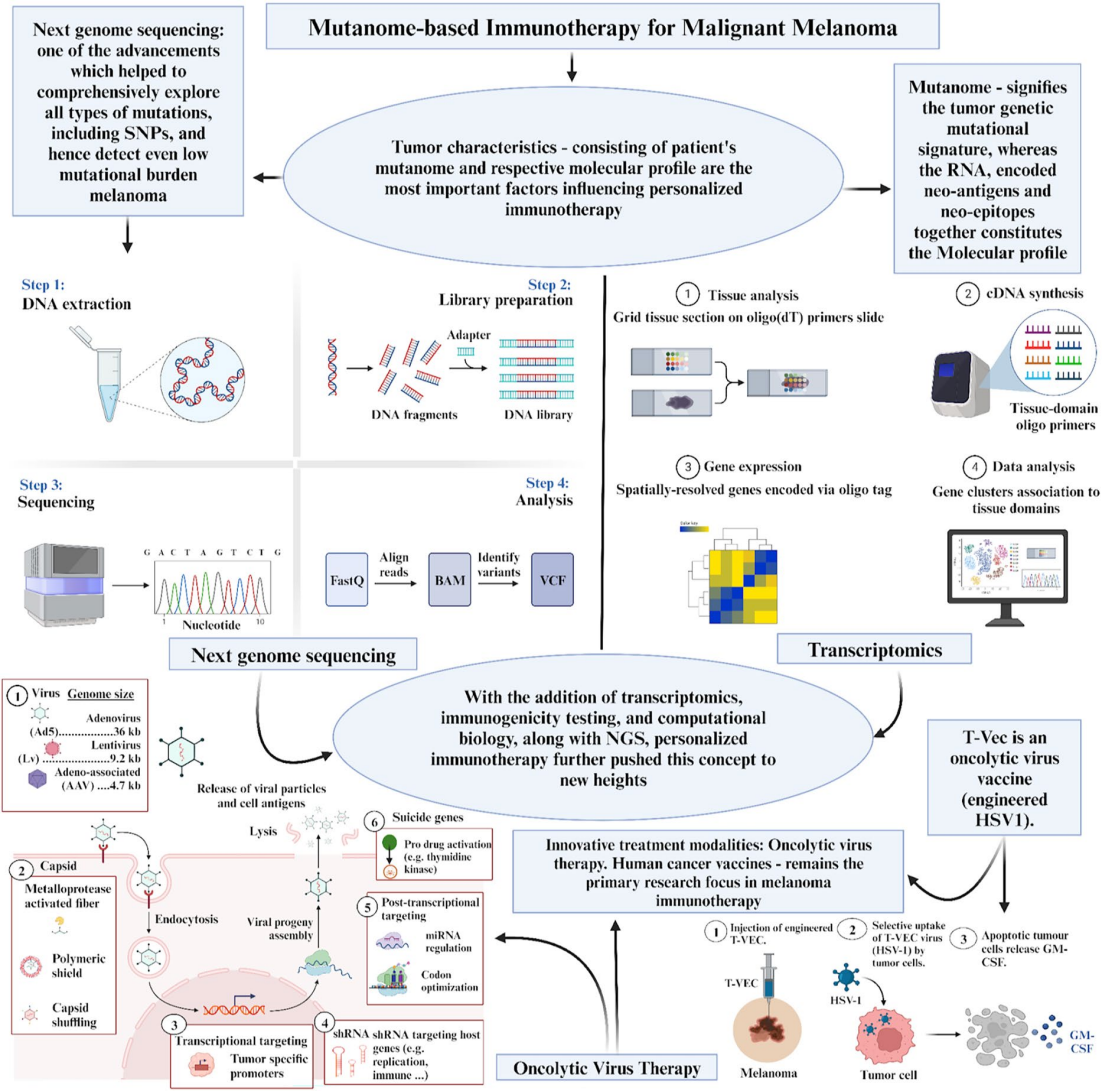
Overview of mutanome-based immunotherapy for malignant melanoma. DNA, deoxyribonucleic acid; RNA, ribonucleic acid; T-VEC, talimogene laherparepvec; GM-CSF, granulocyte macrophage colony stimulating factor; HSV-1, herpes simplex virus-1; NGS, Next Genome Sequencing; BAM, binary alignment map; VCF, variant cell format; SNP, single nucleotide polymorphism; shRNA, small hairpin RNA; cDNA, complementary DNA

Individualisation of melanoma immunotherapy represents a shifting paradigm in the field of oncology towards personalised medicine [7, 56, 57]. This transformation relies on various factors such as biomarker expression [58–64], immune system profiling [65], tumour microenvironment [66], patients' well-being [67, 68], and preferences [69]. However, tumour characteristics, which encompass the patient's mutanome and respective molecular profile, are the most important factor. The role of mutanomes in immunotherapy for malignant melanoma is summarised in Fig. 2. This holds a promising and powerful tool, as most melanoma mutations are unique and rarely shared, even among the same type [9, 70].

The “mutanome” or “mutation-genome” reflects the tumour genetic mutational signature [71], while the molecular profile includes the ribonucleic acids (RNAs) [72], encoded neoantigens [73, 74], and neo-epitopes [15]. This concept emerged thanks to sequencing technologies, especially after publishing the first complete set of mutations in *Saccharomyces cerevisiae* yeast in 2002 [75]. One of the pioneering studies, which was done by Krauthammer and his team, was the first to unveil melanomas' mutational landscape using exosome sequencing [76].

As time progressed, significant advancements in sequencing technologies propelled us from traditional and exosome-only sequencing to embracing more sophisticated approaches, such as whole genome and next-generation sequencing [77]. These advancements have allowed for a more comprehensive exploration of all types of mutations, ranging from single nucleotide polymorphisms (SNPs) and insertions to deletions and frameshifts, regardless of their effect as driver or passenger mutations and irrespective of whether they occur in coding or non-coding regions [77, 78]. This way, we can catch mutations even with low mutational burden melanomas [79, 80].

Moreover, the integration of additional fields such as transcriptomics, immunogenicity testing [81], and computational biology pushed the concept to its extreme limits [82–84]. This enabled us to leverage individualisation by decoding the patient's tumour mutanome using NGS according to the health human genome atlas, predicting neoantigens [85], and identifying epitopes with strong human leukocyte antigen (HLA) binding affinity [83, 86]. That precious information can later be used in different types of immunotherapies.

In the context of adoptive cell therapy, research utilising this knowledge showed that tumour-infiltrating lymphocytes (TIL) prepared based on predicted neoantigens and neo-epitopes exhibited enhanced T cell expansion and response [87, 88]. But like other passive immunotherapies, despite their potential benefits, they lack long-term effectiveness due to challenges like T cell specificity loss [89] and research still trying to solve them [90].

A multimodal immunotherapy that makes use of both passive and active approaches is oncolytic virus therapy. Studies demonstrated that genetically modified viruses, like herpes [91], adenoma, and vaccinia, have the ability to directly lyse virus-infected melanoma cells and release tumour neoantigens, stimulating anti-tumour immunity [2, 93]. In recent studies, scientists have further enhanced active immunotherapy by coating viruses with predicted tumour neoantigens, Peptide-coated Conditionally Replicating Adenovirus (PeptiCRAd) [94]. This innovative technique holds great promise for future research.

However, vaccines continue to remain the primary research focus of active melanoma immunotherapy [95–97]. Mutanome-based individualisation approaches have been employed to develop on-demand vaccine manufacturing pipelines and conduct thorough testing. Various vaccine types, including peptide, RNA, and dendritic cell vaccines, have been studied.

For instance, autologous dendritic cells loaded ex vivo with patient-specific neoantigens demonstrated good tolerance and an increase in the breadth and diversity of T cell responses [98–100]. Subsequently, custom messenger ribonucleic acid (mRNA) liposomal vaccines capable of neoantigen encoding were developed and tested, resulting in the expansion of preexisting T cells and the induction of new T cell responses against the neo-epitopes [101–104]. Other studies explored a custom peptide vaccine synthesis approach using patients' neoantigen structures [105, 106].

This approach to melanoma treatment ensures that the treatment is tailored to the individual profile, maximising the chances of a successful immune response and reducing the risk of treatment resistance. This can also directly or indirectly target cancer cells and kill them. Overall, the integration of mutanome knowledge in individualised immunotherapy holds promise for revolutionising melanoma treatment, offering patients the potential for better responses, prolonged remissions, and a step closer to achieving the goal of precision oncology.

Despite our advancing knowledge of the mutanome, several limitations remain when implementing this research for individualised immunotherapy of malignant melanoma. One of the biggest limitations is the impact physiological differences in the body have on the absorption, distribution, metabolism, and elimination of drugs [107–109]. Immune checkpoint inhibitors (ICI) have revolutionised the treatment of malignant melanoma; however, the response rate is approximately one-third [110–112]. A lack of research into the pharmacokinetic responses of ethnicity, age, sex, and disease stage, however, limits the effectiveness of individualised immunotherapy [113]. Targeting this area of research remains challenging due to the large genetic variations that exist within these subpopulations [114]. Effectively targeting and utilising such data could allow individualised immunotherapy for malignant melanoma to reach its full potential [70].

## AI and cancer immunotherapy

### AI techniques of importance in cancer immunotherapy

In the field of cancer treatment, immunotherapy has made significant advancements and is now widely used. However, a challenge that has arisen is the identification of suitable individuals who can benefit from this therapy and who should receive it. To address this challenge, AI has been developed to aid in performing tasks that typically require human intelligence. These tasks include interpretation of language, perception of visual materials, and decision-making [115]. The utilisation of AI technologies has resulted in enhanced precision and effectiveness in the diagnosis and prediction of cancer treatment responses. AI has enabled the classification of patients into two groups: those who will respond positively to cancer immunotherapy and those who will not, thereby ensuring that only suitable patients receive the treatment [116]. With the aid of neural-based models, the tumour immune microenvironment of solid tumours such as colorectal, breast, lung, and pancreatic cancer, which plays a crucial role in patients' responses to cancer immunotherapy, has been accurately characterised by integrating RNA sequencing (RNA-Seq) and imaging data in a clinical setting [117].

Currently, numerous research groups and companies are dedicated to creating programmes that can enhance the efficiency, precision, and affordability of cancer screening. By acting as a supplementary visual aid, AI can aid medical professionals in identifying and diagnosing cancer in images with greater precision than would be possible otherwise. This results in improved accuracy and, consequently, insight for patients [117]. The application of deep learning (DL) methods enables the precise and automated identification of changes in tumour size and gene status, which can serve as an assisting tool for monitoring the efficacy of immunotherapy [115].

As biotechnology continues to develop and our understanding of the molecular mechanisms of tumours expands, immunotherapy has become an effective method of training the immune system to recognise and target specific cancer cells. This treatment modality can enhance the immune cells' ability to identify and eliminate cancer cells while also providing the body with supplementary components to augment the immune response. There are different types of cancer immunotherapy available, including targeted antibodies, cancer vaccines, adoptive cell transfer, tumour-infecting viruses, checkpoint inhibitors, cytokines, and adjuvants. In the prediction of immunotherapy responses, AI has been employed in the evaluation of immune signatures, medical imaging, and histologic analysis [117].

### Current application of AI in the individualisation of cancer immunotherapy

The utilisation of AI, a cutting-edge technology, has made it possible to provide personalised treatment to patients with tumours by automating the prediction of the effects of tumour immunotherapy through the construction of models [118]. The use of AI in immunotherapy is concentrated on three main themes. The first theme concerns tumour neoantigens, which form the foundation of immunotherapy. A key unresolved issue in this area is the rapid and precise prediction of immunogenic tumour antigens using AI, which would minimise the need for experimental screening and validation [119]. Machine learning (ML) techniques have the potential to identify the factors that determine tumour immunogenicity and the peptides presented by major histocompatibility complex class I (MHC-I), which can be utilised to assess neoantigen binding and/or treatment response predictions in cancer immunotherapy [115]. Artificial neural networks enable the observation of tumour antigen T cell epitopes in patients with melanoma, which can be utilised for personalised cancer immunotherapy [117]. The second theme of AI application in immunotherapy pertains to the scope for improvement in tumour therapeutic monoclonal antibodies, despite their notable success. This has spurred much innovation in antibody design, with AI-augmented antibodies holding immense potential for further advancements in cancer treatment. The advent of DL has opened up new avenues for therapeutic antibody design, including the prediction of structure, screening for target binding, affinity maturation, and pharmaceutical property prediction.

The third theme pertains to the challenges associated with predicting the response to immunotherapy. This includes the identification of patients who are most likely to respond to immunotherapy using multimodal and multi-scale biomarkers, as well as the characterisation of the tumour immune microenvironment [119]. AI-based techniques like imaging and histopathology analysis both ML-based and DL-based approaches have demonstrated efficacy in interpreting tumour microenvironment (TME) in combination with immunohistochemistry. These methods reveal disparities in the expression and localisation of biomarkers among various histological subtypes, which can be leveraged to predict responses to immunotherapies or other targeted therapies [115].

To predict the effectiveness of immunotherapy using AI, a general approach involves creating a training cohort and a validation cohort. The multi-scale medical data from the training cohort are collected, filtered, segmented, and features extracted and selected. This data is then used to train and model AI. The validation cohort is used to verify the results of the AI's learning. The multi-scale medical data may include genomics, proteomics, pathological tissue, computed tomography / magnetic resonance (CT/MR) imaging, and more. The goal is for the AI to predict whether a patient will benefit from immunotherapy or suggest further evaluation, such as whole genome sequencing. Additionally, AI can predict which immunotherapy drug will be most effective for the patient. This approach can improve the accuracy of immunotherapy treatment and potentially lead to better patient outcomes [118].

Radiomics is an emerging AI technique that is gaining increasing attention in cancer management. It is an algorithm-based method that extracts patterns from images obtained from computed tomography, magnetic resonance imaging, positron emission tomography or a combination of two of these [120, 121]. These patterns serve as the basis for response rate monitoring [122], individualisation of therapy [122], risk stratification [121, 123], survival analysis [123], metastatic capability predictions [121, 124] and patient monitoring [122]. In the individualisation of therapy, this is especially useful as it can discern little differences in obtained images, thereby forming patterns that can be used in correlation generation, thus influencing therapy choices. One of such is its application as a predictive signature generator for better correlation with immune markers. CD8^+^ expression in melanoma was found to be inversely proportional to the mean of positive pixel (MPP) and standard deviation (SD) using radiomics which also correlates with prognostic outcomes in patients [125]. It has similarly been applied in signature–immune marker correlations in other types of cancers like non-small-cell lung [126] and renal cancers [127]. Furthermore, signature correlations have also been used in evaluating survival in melanoma patients treated with pembrolizumab [123].

### Advantages and limitations of the use of AI in cancer immunotherapy

AI has emerged as a highly advanced tool in the field of computer-assisted cancer immunotherapy. As clinical data and AI methodologies continue to advance, AI has the potential to play an even greater role in predicting immunotherapy responses. One of the greatest strengths of AI is its ability to learn from large sets of data and identify patterns that can be applied to specific tasks, such as mutation annotation or diagnosis [128].

The incorporation of AI in cancer immunotherapy has been recognised as a developing computer-assisted approach that can enhance the predictive abilities and functional roles of personalised therapy. Nonetheless, there are discrepancies in the application of AI techniques for widespread use in clinical practice. AI-based algorithms have the potential to be a promising strategy for optimising individualised immunotherapy and ultimately improving the healthcare quality and prognosis of patients [115].

The application of AI in cancer immunotherapy has demonstrated some limitations. These include a shortage of available data, data biases, insufficient data sharing, a lack of code sharing, and difficulties in interpreting the models. Also, there is a gap between the ease of gathering data from various platforms and the ease of access by external agencies for independent use, especially for private or controlled-access datasets. The absence of data sharing hinders the effective validation of AI models across multiple medical centres. Additionally, the variability of data presents a significant challenge in implementing DL for immunotherapy, whereby incongruities in data batches and quality issues often lead to unsuccessful external validation [119].

The intricate nature of predicting immunotherapy outcomes necessitates collaboration between scientific researchers, enterprises, and clinicians to construct databases and establish industry standards. This collaborative effort should aim to eliminate technical obstacles and foster the development of AI-assisted systems that can precisely identify the target population for immunotherapy, accurately forecast treatment efficacy and prognosis, and promote the implementation of AI-assisted treatment while earning the trust of both physicians and patients [118].

## AI in refining mutanome-based immunotherapy of malignant melanoma

**Fig. 3:**
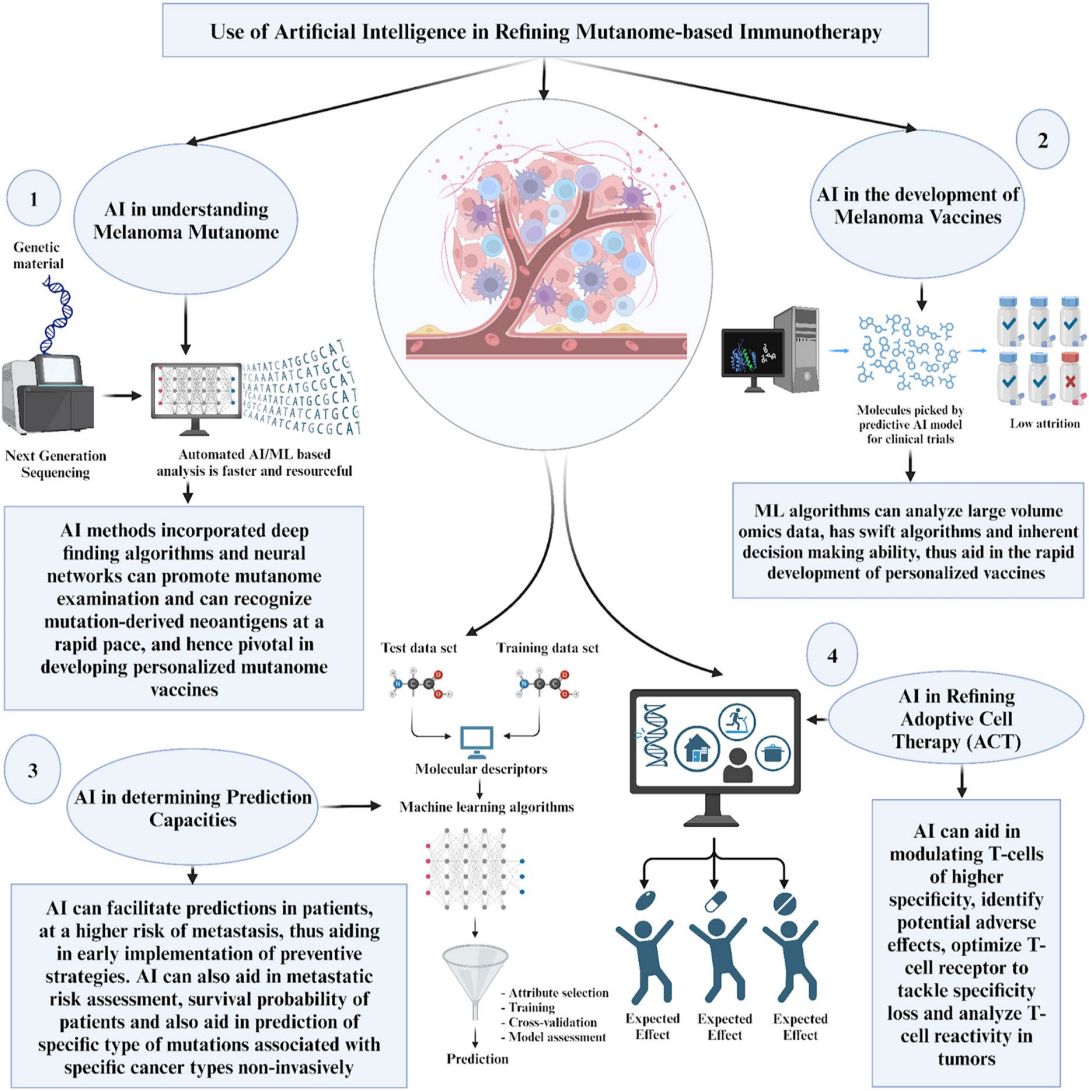
Application of AI refining in mutanome-based immunotherapy. ACT, adoptive cell therapy; AI, artificial intelligence; ML, machine learning

AI is a tool that can potentially change outcomes in malignant melanoma. With advances in AI, the sequencing of melanoma mutations quickly, the development of individualised vaccines, the determination of the response rate to individualised immunotherapy, patient stratification based on predicted outcomes, and modifying the use of adoptive cell therapy can be refined to meet the increasing needs of melanoma patients. The use of AI in refining mutanome-based immunotherapy is summarised in Fig. 3.

### AI in understanding melanoma mutanome

AI has the potential to advance comprehension of the melanoma mutanome and its significance for immunotherapy. Melanoma harbours an exceptionally high mutational burden, which produces tumour-particular neoantigens that can be targeted by the immune system [21, 130]. Nevertheless, completely exploiting the mutanome necessitates the identification of immunogenic mutations from whole genome and transcriptome data [131]. AI methods employing deep finding algorithms can promote mutanome examination, and this has been used to advance rapid technology-based identification and validation of individual mutanomes by individualised vaccines against cancer (IVAC) in the individualisation of immunotherapy for malignant melanoma [132]. Also, neural networks can recognise mutation-derived neoantigens by incorporating genomic, epigenomic, and immunogenicity information at an unprecedented scale and swiftness [133]. This will empower exhaustive mining of the melanoma mutanome to develop personalised mutanome vaccines [134]. AI can also uncover mutations related to immunotherapy response or resistance, guiding individual categorisation and combination tactics [129].

By accelerating mutanome profiling, AI has the potential to boost comprehension of how the mutational landscape influences immunotherapy efficacy in melanoma. This may reveal new pathways to conquer resistance by targeting special mutational signatures [135]. AI-driven multi-omic relationships with clinical outcomes could also supply insights into optimising mutanome-focused methods through rational drug combinations impacting ribosome biogenesis or epigenetics [136, 137]. Thus, AI is well-positioned to revolutionise understanding of the melanoma mutanome's benefits for immunotherapy through thorough assessment of its tumour-particular abnormalities.

### AI in the development of melanoma vaccines

Personalising melanoma vaccines based on mutanomes is an up-and-coming application of AI [95–97]. With machine learning algorithms able to learn from large amounts of omics data and make inferences that can be applied to new situations, the identification of neoantigens can be optimised, leading to the development of individualised vaccines for different mutational variants [128]. Aside from this, machine learning can streamline vaccines suited to individual immune profiles and hasten the development of large amounts of vaccines for different individuals in a short period of time due to swift algorithms and inherent decision-making capabilities [128]. An mRNA vaccine, mRNA-4157, in a phase 3 clinical trial for melanoma by Moderna and Merck utilises proprietary algorithms in the identification of mutanomes as targets of the vaccine [138]. Advancements in these algorithms are also predicted to shorten the production time from 6 weeks to 30 days, thus increasing the turnaround time, which is one of the major issues identified with the individualisation of immunotherapy [138]. As is known, neoantigens result from mutations in tumours, which can vary among melanoma patients [56]. The identification of immunogenic neoantigens has been challenging so far [139]. However, AI advancements in next-generation sequencing (NGS) have made it possible to identify neoantigens, which are ideal vaccine targets [56]. An AI tool developed by Evaxion (EVX) named Pioneer Technology has been used to identify specific neoantigens for individualised melanoma vaccines like EVX-01 and EVX-02, which are novel molecules at various stages of clinical trials [140].

### AI in refining adoptive cell therapy immunotherapy option

In addition to vaccines, AI can refine the adoptive cell therapy (ACT) immunotherapy option. It is known that melanoma mutations are unique and rarely shared [9, 70]. Thus, AI can build on this knowledge to enhance the modulations of T cells, having greater specificity for individual mutations. Also, with deep learning algorithms, AI can simulate what happens when ACT is used, thus limiting resource waste and identifying major lapses and potential adverse effects early on in the drug discovery process [141]. A major problem with ACT is T cell specificity loss, which can be optimised by T cell receptor (TCR) deep sequencing. However, TCRs of significance are rare to come across. With AI, TCR can easily be identified compared to previous experiences via machine learning algorithms that can predict TCR–target interactions specific to every individual [89]. Another issue with ACT is that T cells cannot recognise all mutanomes in tumours. Rather than using peptide-binding algorithms to identify immunogenic mutations, advancements in predictive algorithms have made it possible for minigenes to analyse T cell reactivity in tumours, thus making it possible to develop novel ACTs that recognise individual neoantigens [142].

### AI in determining prediction capacities

AI presents an advancing approach that can achieve things that were previously deemed resource-intensive in melanoma. This can help improve prediction capacities, thus increasing the drug discovery pipeline efficiency [143]. In particular, AI can improve predictions in patients that are at a higher risk of metastasis based on their mutanome [141], thus allowing for early preventive measures that can increase patient survival rates. Different melanoma mutanomes are associated with varying levels of serum biomarkers [144]. Some predictive biomarkers, like dermcidin, interferon-gamma, interleukin-4, and granulocyte macrophage colony stimulating factor (GM-CSF), are associated with metastatic melanoma in early-stage patients [141]. Using an AI algorithm to streamline metastatic risk assessment can help improve immunotherapy options that will best reduce the risk of metastasis at an early stage. Machine learning can also increase the speed of determining the probability of survival in melanoma patients. This was demonstrated in research where a combination of machine learning and radiomics was used to assess the survival rates of advanced melanoma patients treated with the immune checkpoint blocker pembrolizumab [123]. This approach can benefit from machine learning’s ability to automate how lesions are identified and segmented in melanoma. AI has also been used to predict the specific type of mutations that initiated a particular cancer in an individual via a noninvasive method [145]. The detailing of the *BRAF* mutation underlying the melanoma brain metastasis using machine learning-assisted radiomics technique was achieved in contrast to the norm where tissue biopsy is required to determine the genetic aspect of brain metastasis [145]. This noninvasive approach presents a novel technique that can be utilised to predict the exact mutanome in melanoma, thus facilitating better immunotherapy selection [145]. However, this method cannot predict the development of metastasis in specific patients. Table 2 provides a summary of the potential impact of AI on the enhancement of mutanome-based immunotherapy for malignant melanoma.

**Table 2:** Summary of the role of AI in refining mutanome-based immunotherapy of malignant melanoma

Advantages	Description
Enhance understanding of melanoma mutanome [120–124, 129, 132, 133, 135–137]	• Deep finding algorithms can promote mutanome examination, that is used to advance rapid technology-based identification and validation of individual mutanomes by IVAC
	• Neural networks recognise mutation-derived neoantigens by incorporating genomic, epigenomic, and immunogenicity information at an unprecedented scale and swiftness
	• Uncover mutations related to immunotherapy response or resistance, guiding individual categorisation and combination tactics
	• Potential to reveal new pathways to conquer resistance by targeting special mutational signatures
	• Supply insights into optimising mutanome-focused methods through rational drug combinations impacting ribosome biogenesis or epigenetics
	• Radiomics extract patterns from imaging modalities like CT, MRI, and PET
	• Patterns derived from radiomics serve as a basis for response rate monitoring, risk stratification, survival analysis, metastatic capability predictions, and patient monitoring
	• In individualised therapy, radiomics discerns subtle differences in images, forming patterns influencing therapy choices
Facilitate the development of melanoma vaccines [95–97, 125, 128]	• Personalised melanoma vaccines
	• Radiomics contributes to individualised therapy by generating predictive signatures
	• Optimised identification of neoantigens, leading to the development of individualised vaccines for different mutational variants
	• Streamline vaccines suited to individual immune profiles
	• Hasten the development of large amounts of vaccines for individuals in a short period of time
Refining adoptive cell therapy immunotherapy option [9, 70, 89, 125–127, 141, 142]	• Refine ACT
	• Enhance the modulation of T cells, having greater specificity for individual mutations
	• Limit resource waste and identify major lapses and potential adverse effects early through simulation
	• Mitigate T cell specificity loss, optimised by TCR deep sequencing
	• Development of novel ACTs that recognise individual neoantigens, enabled by advancements in predictive algorithms for minigenes to analyse T cell reactivity in tumours
	• Application in signature-immune marker correlations extends to other cancers like non-small-cell lung and renal cancers
Determine prediction capacities [123, 141, 143, 145]	• Improve prediction capacity, thus increasing drug discovery pipeline efficiency
	• Predict specific type of mutations that initiate cancer in an individual via a noninvasive method (machine learning-assisted radiomics technique)
	• Improve predictions in patients at higher risk of metastasis based on their mutanome
	• Streamline metastatic risk assessment
	• Allows for early preventive measures that can increase patient survival rates
	• Automate the identification and segmentation of lesions in melanoma
	• Radiomics serve as a predictive signature generator, aiding in better correlation with immune markers
	• Signature correlations have been utilised in evaluating survival in melanoma patients treated with pembrolizumab

ACT, adoptive cell therapy; AI, artificial intelligence; IVAC, Individualised vaccines against cancer; TCR, T cell receptor

### Future prospects and potential limitations of AI in advancing and refining mutanome-based immunotherapy for malignant melanoma

AI is a powerful tool that can change the future management and outcomes associated with malignant melanoma. In light of rising concerns about the development of resistance to available immunotherapy options, exploring the mutanome-based immunotherapy approach refined by AI is gaining traction. Limited research has been carried out on how understanding individual mutations can benefit therapy outcomes due to the diverse nature of the mutations underlying the development of melanomas [9, 70]. Also, the use of AI in melanoma management is not without drawbacks. Notably, imprecisions in AI’s ability to adequately detect lesions in people outside the dataset used in developing the AI algorithm have been cited [146], thus raising concerns about AI’s inference applicability in a larger population [146]. However, AI still remains a game changer that can effectively turn the tide on melanoma management.

A futuristic utility of AI is its ability to swiftly through large sets of mutanomes in a short time. One major problem often cited in the development of individualised immunotherapy is the time and resource intensiveness of sequencing individual mutanomes and modulating immunotherapy options specific to the mutanomes. ML can process large amounts of data in a relatively short time, carry out gene-treatment pairing for best fit, determine the chances of toxicity and efficacy, and also use this data as a pattern for future predictions [143].

Exploring AI to improve individualised immunotherapy options based on mutanomes remains an aspect of the cancer drug discovery process requiring much attention. The growing resistance to multiple immunotherapies available for melanoma continues to dash the hopes of discovering immunotherapy ignited in the scientific world. Worse still, other therapy options like chemotherapy are ineffective in achieving the cure rates obtained from immunotherapies. Thus, it is important to improve and facilitate research that seeks to enhance the application of AI in individualising therapy best suited for the specific genetic mutations in every patient. With improvements in technologies and newer AI algorithms developing, individualised immunotherapy is becoming a possibility. This will help increase the efficiency of the drug discovery process, reduce adverse drug events, and increase survival rates in melanoma patients.

While AI shows promise for optimising mutanome-based immunotherapy, certain limitations must be addressed. Accurately predicting immunogenic neoantigens from tumour sequencing data remains challenging due to tumour heterogeneity and the complexity of antigen presentation [21, 137]. DL models require vast amounts of high-quality immunogenomic training data, which are difficult to obtain, potentially limiting generalisability [131, 133].

Additional barriers include the dynamic interplay between mutations, epigenetic modifications, and cellular signalling pathways influencing immunogenicity, which are challenging to fully incorporate into static AI models [135, 137]. Mutational signatures associated with endogenous and exogenous DNA damage involve complex biological processes not easily defined by current machine learning algorithms [135]. There are also ethical concerns around explaining “black box” AI predictions to patients and difficulties validating models using prospective clinical trial data [129, 131].

Overcoming these limitations requires multidisciplinary collaborations between clinicians, immunologists, geneticists, and AI specialists. Larger pan-cancer immunogenomic databases with linked multi-omic profiles and treatment outcomes could improve generalisability but represent a major undertaking [133]. Combining unsupervised and supervised machine learning with mechanistic modelling may help capture tumour biology dynamics not evident from bulk sequencing alone [133, 137]. With refinements, AI has the potential to optimise mutanome-based therapies if technical challenges around data, modelling complexity, and clinical integration are addressed.

## Conclusion

AI is a tool with vast potential in melanoma, as demonstrated by numerous studies on how to optimise its use to simplify management approaches. Although it is not without lapses, its application in rapidly sequencing mutanomes to enhance the ease of individualising therapy in all stages of melanoma is revolutionary. Thus, exploring AI to refine mutanome-based individualisation of therapy can strengthen current predictions of response and toxicity in melanoma patients at all stages. Owing to its robustness, it can also be used to predict the kind of mutation underlying a cancer type, thus easing the ease of patient stratification for immunotherapy and helping future prevention of metastasis. However, more research is required to address the shortcomings of AI in its multiple distinguishing capabilities, large-scale application, and data porosity in order to aid its future outcomes in melanoma.

## Data Availability

No data available.

## Abbreviations

CTLA-4: Cytotoxic T-lymphocyte associated protein-4
PD-1: Programmed cell death protein 1
TCR: T cell receptor
PDL-1: Programmed death ligand 1
MHC: Major histocompatibility complex
DC: Dendritic cell
BRAF: V-raf murine sarcoma viral oncogene homolog B1
MEK: Mitogen-activated protein kinase
ERK: Extracellular signal-regulated kinase
RAF: Rapidly activated fibrosarcoma
RAS: Rat sarcoma
RTK: Receptor tyrosine kinase
GTP: Guanosine triphosphate
NRAS: Neuroblastoma ras viral oncogene homolog
PTEN: Phosphatase and tensin homolog
TP53: Tumour protein 53
BRCA: Breast cancer gene
CDK: Cyclin dependent kinase
DNA: Deoxyribonucleic acid
RNA: Ribonucleic acid
T-VEC: Talimogene laherparepvec
GM-CSF: Granulocyte macrophage colony stimulating factor
HSV-1: Herpes simplex virus-1; NGS: Next Genome Sequencing
BAM: Binary alignment map
VCF: Variant cell format
c-Kit: Tyrosine-protein kinase kit
SNP: Single nucleotide polymorphism
shRNA: Small hairpin RNA
cDNA: Complementary DNA
ACT: Adoptive cell therapy
AI: Artificial Intelligence
ML: Machine Learning
IVAC: Individualised vaccines against cancer
CDKN2A: Cyclin dependent kinase inhibitor 2A
mRNA: Messenger RNA; UV: Ultra violet
AIDS: Acquired immunodeficiency syndrome
NCCN: National Comprehensive Cancer Network
CSD: Chronic sun damage
SSM: Superficial spreading melanoma
NM: Nodular melanoma
ABCDE: Asymmetry, border, colour, diameter, and evolving
AM: Acral melanoma
HLA: Human leukocyte antigen
TIL: Tumour-infiltrating lymphocyte
EVX: Vaxion
PeptiCRAD: Peptide-coated Conditionally Replicating Adenovirus
ICI: Immune checkpoint inhibitors
RNA Seq: RNA sequencing
DL: Deep learning
TME: Tumour microenvironment
CT: Computed tomography
MR: Magnetic resonance
MPP: Mean of positive pixel
SD: Standard deviation

## Declarations

Ethics approval and consent to participate.

Not applicable.
